# Towards adaptive deep brain stimulation for freezing of gait

**DOI:** 10.1093/brain/awac172

**Published:** 2022-07-15

**Authors:** Huiling Tan

**Affiliations:** Medical Research Council Brain Network Dynamics Unit, Nuffield Department of Clinical Neurosciences, University of Oxford, Oxford, UK

## Abstract

This scientific commentary refers to ‘Cortical phase-amplitude coupling is key to the occurrence and treatment of freezing of gait’ by Yin *et al.* (https://doi.org/10.1093/brain/awac121).

**This scientific commentary refers to ‘Cortical phase-amplitude coupling is key to the occurrence and treatment of freezing of gait’ by Yin *et al.* (https://doi.org/10.1093/brain/awac121).**

Freezing of gait (FoG) is a debilitating symptom affecting over half of patients with advanced Parkinson’s disease. Yet, the neurobiological basis of FoG is poorly understood, and the available therapies are unsatisfactory. One of the key therapies for Parkinson’s disease, deep brain stimulation (DBS), is currently undergoing a period of intense innovation. So-called ‘adaptive’ DBS automatically adjusts the stimulation in response to fluctuating biomarkers such as neural synchrony to increase the efficacy of DBS for suppressing bradykinesia and rigidity while reducing side effects.^1^

**Figure 1:**
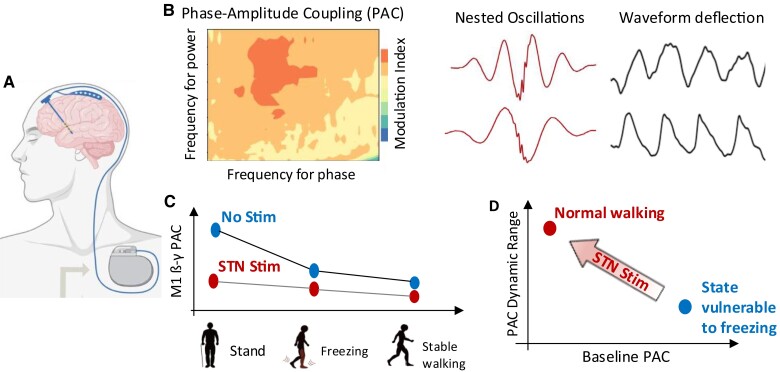
**Schematic showing the key findings of Yin and colleagues.^2^** (**A**) Potentials from primary motor cortex were recorded from patients with Parkinson’s disease undergoing surgery for DBS. (**B**) Beta-gamma PAC in the motor cortex was observed, which can arise from either nested oscillations, or deflected waveforms in the beta frequency bands, or both (adapted from Cole *et al.*^3^ and Vaz *et al.*^4^). (**C**) PAC in motor cortex decreased during walking compared to standing, but temporarily increased during states vulnerable to freezing, compared with normal walking. (**D**) STN stimulation reduced the baseline PAC and increased the dynamic range of PAC for normal functioning.

This success has raised the question of whether DBS can also be optimized to treat FoG. Achieving this goal will require identifying reliable biomarkers and understanding more completely the neural signatures underlying this phenomenon. In this issue of *Brain*, Yin and colleagues^2^ make progress towards this goal by identifying increased beta-gamma phase-amplitude coupling (PAC) in the motor cortex during freezing episodes compared to normal walking in patients with Parkinson’s disease. In addition, they show that DBS of the subthalamic nucleus (STN) reduces this coupling along with freezing episodes.

A variety of cognitive and affective challenges can trigger freezing episodes in Parkinson’s disease, suggesting a cortical contribution to the pathogenesis of FoG. However, the neurophysiological characteristics of FoG within the motor cortex and how they respond to cognitive overload and DBS remain largely unknown. In this study, 16 patients with Parkinson’s disease undergoing STN DBS surgery were temporarily implanted with a subdural electrocorticography (ECoG) grid placed above the right primary motor cortex (M1). The ECoG lead was externalized for an average of 8 days prior to placement of the pulse generator. This allowed the authors to record local field potentials (LFPs) from M1 during a walking task with concomitant recordings of gait kinematics using a 3D motion tracking system.

The authors detected higher beta-gamma PAC in M1 during walking trials with freezing episodes than trials without. Though with very limited spatial coverage, the investigators showed that the increased PAC was specific to the primary motor cortex and was not observed in the postcentral gyrus (S1). Electrical stimulation delivered to the STN reduced PAC in M1 and simultaneously improved freezing. The authors concluded that elevated beta-gamma PAC in the primary motor cortex indicates a higher probability of freezing, and that therapeutic DBS alleviates freezing by both decoupling cortical oscillations and increasing the dynamic range of the PAC for normal functioning, thus enhancing cortical resistance to abnormal coupling (Fig. 1). These novel findings raise several questions.

##  

### What is the neural mechanism underlying elevated phase-amplitude coupling and how is it related to freezing?

Statistical PAC quantifies how much the phase of a low frequency oscillation modulates the amplitude of a high frequency oscillation. At least two different neural mechanisms can lead to increased PAC: (i) the interactions between low and high frequency oscillations; and (ii) repeated sharp or non-sinusoidal deflections in the waveforms of the low frequency oscillation.^3,4^ Both are forms of elevated synchrony indicative of reduced cortical information processing capability, and therefore could be relevant to the pathology of FoG. However, the two interpretations may have important implications for the mechanisms of PAC and its pathophysiological significance. Detailed time-domain waveform analyses can offer more insights into the neural mechanism of PAC.^3,4^

Yin and colleagues^2^ showed that cortical PAC during walking was moderately correlated with the beta band waveform asymmetries, suggesting that the waveform asymmetry contributed to the PAC. However, this is unlikely to be the sole explanation for elevated PAC. More likely, nested oscillations and changes in β waveforms co-occur during freezing.

Elevated PAC due to nested oscillations may reflect a loss of physiological segregation of distinct feedback loops within the basal-ganglia-thalamocortical (BGTC) circuit, leading to coupling between otherwise distinct circuits. Increased synchronization in the β-frequency range in the BGTC circuit is a hallmark of Parkinson’s disease. Synchronization of basal ganglia spiking is likely to promote a rhythmic pattern of spike timing in target cortical areas, which can be detected as a contributor to broadband-γ spectral power and manifests as exaggerated coupling of the β-phase to broadband-γ activity.^3^ In patients with freezing, activity in the low β frequency band (12–22 Hz) was significantly enhanced in the STN during walking compared to patients without freezing.^5^ Transient increases in STN multi-unit activity in the theta and beta frequency bands were also observed before the onset of freezing.^6^ Simultaneous recordings of LFPs from the cortex and the STN could show if the increased cortical β-γ PAC in freezing trials is a correlate of increased β in the STN or an independent phenomenon.

On the other hand, elevated PAC due to repeated deflections of the beta band waveform may reflect local synchrony of excitatory input currents onto cortical pyramidal neurons. The exact waveform change may reflect the temporal organisation of the synaptic inputs. Cortical PAC during walking predicted freezing in Parkinson’s disease and correlated with waveform asymmetry (the ratio of the rising versus falling steepness).^2^ However, cortical PAC during rest has also been shown to correlate with other parkinsonian symptoms including rigidity and bradykinesia, and was better explained by the asymmetry in extrema sharpness.^3^ Further analysis of the cycle-by-cycle waveforms and preferred phase of high frequency activity may reveal differences in the temporal organisation of synaptic inputs and further help to differentiate rest, freezing and standing.

### Can cortical phase-amplitude coupling serve as a biomarker for freezing of gait?

Titrating the timing and intensity of stimulation according to biomarkers that capture current clinical state has great potential to improve efficacy, reduce side effects, and decrease the cost of treatment. To achieve this goal, identifying appropriate feedback signals that faithfully reflect the clinical state and can be measured with minimal extra interventions is crucial.^1^

Chronic recording of LFPs using subdural cortical grids has been shown to be feasible and safe over a one-year period.^7^ Here, Yin and colleagues showed that β-γ PAC can be reliably calculated in a 10-s window. However, time-domain waveform analyses, such as the waveform symmetry, which correlated with PAC, can be calculated for individual cycles and require less computational power. Thus, these waveform parameters could potentially provide an instantaneous measure of clinical state so that therapy does not lag impairment.

Apart from feasibility, we also need to evaluate the sensitivity and specificity of PAC as a correlate or predictor for FoG. Yin and colleagues showed that elevated PAC was not due to dual tasking or reduced movement velocity. However, the highest PAC was observed during standing. Therefore, a single threshold-based method would not be sufficient to differentiate freezing from both standing with high PAC and normal walking with low PAC. Since excessive PAC was also observed during non-freezing episodes in FoG trials, the temporal relationship between FoG and PAC needs to be further investigated. In addition, the authors reported that at a similar PAC level, trials with STN stimulation had significantly lower probability of containing freezing episodes than no-stimulation trials, suggesting that the exact relationship between PAC and freezing depends on the stimulation. Previous observations of correlations between enhanced PAC over broad cortical regions and other parkinsonian symptoms such as bradykinesia,^3,8^ highlight the fact that further studies are required to test the specificity of temporally enhanced PAC in M1 as a biomarker for freezing.

### How to optimize deep brain stimulation to better treat freezing of gait?

Once a biomarker is detected, one way to improve DBS is to change the stimulation to a predefined pattern that is more suitable for the specific clinical state. Low-frequency stimulation (generally 60 Hz or 80 Hz) may help some patients with axial symptoms. However in this study,^2^ 60 Hz stimulation did not reduce freezing episodes compared to 130 Hz stimulation. Stimulating the substantia nigra pars reticulata (SNr) has been shown to improve temporal parameters of gait^9^ and combining low-frequency SNr stimulation and high-frequency STN stimulation may have a synergistic effect for some patients. Recently, Fischer *et al*.^10^ found that alternating stimulation between the left and right STN in a rhythm close to the natural stepping frequency, which resembles the endogenous STN modulation pattern observed during stepping, could entrain patients’ stepping movements. It remains to be tested if this new biomimetic stimulation pattern can facilitate rhythmic movements during free walking and thus reduce freezing.

Another way to improve DBS is to specifically target pathological brain signals by optimizing the precise pattern of stimulation. In this case, the brain signal must not merely correlate with the clinical state, but must also cause it in order to be a legitimate target.^1^ FoG can be triggered by multiple scenarios, including encountering a narrow doorway. If we can understand what causes FoG in response to sensory inputs or increased cognitive demands, we could develop tailored stimulation patterns involving cross-site sensing and stimulation that can modulate local synchrony as well as cross-site functional connectivity. Such an approach has the potential to probe the causal relationship between different neural signatures and freezing, and may also open up new therapeutic avenues.

In summary, to improve DBS for better treatment of FoG, a more detailed understanding of the pathological brain signals underlying this symptom and more research on novel stimulation patterns and targets will be required.
